# Whole-heart computational modelling provides further mechanistic insights into ST-elevation in Brugada syndrome

**DOI:** 10.1016/j.ijcha.2024.101373

**Published:** 2024-03-04

**Authors:** Eike M. Wülfers, Robin Moss, Heiko Lehrmann, Thomas Arentz, Dirk Westermann, Gunnar Seemann, Katja E. Odening, Johannes Steinfurt

**Affiliations:** a Institute for Experimental Cardiovascular Medicine, University Heart Center Freiburg – Bad Krozingen, and Faculty of Medicine, University of Freiburg, Freiburg, Germany; b Department of Physics and Astronomy, Faculty of Sciences, Ghent University, Ghent, Belgium; c Department of Cardiology and Angiology, University Heart Center Freiburg – Bad Krozingen, and Faculty of Medicine, University of Freiburg, Freiburg, Germany; d Translational Cardiology, Department of Cardiology and Institute of Physiology, University Hospital Bern, University of Bern, Switzerland

**Keywords:** Brugada, Depolarization, Repolarization, ST-elevation, Computational modeling

## Abstract

**Background:**

Brugada syndrome (BrS) is characterized by dynamic ST-elevations in right precordial leads and increased risk of ventricular fibrillation and sudden cardiac death. As the mechanism underlying ST-elevation and malignant arrhythmias is controversial computational modeling can aid in exploring the disease mechanism. Thus we aim to test the main competing hypotheses (‘delayed depolarization’ vs. ‘early repolarization’) of BrS in a whole-heart computational model.

**Methods:**

In a 3D whole-heart computational model, delayed epicardial RVOT activation with local conduction delay was simulated by reducing conductivity in the epicardial RVOT. Early repolarization was simulated by instead increasing the transient outward potassium current (I_to_) in the same region. Additionally, a reduction in the fast sodium current (I_Na_) was incorporated in both models.

**Results:**

Delayed depolarization with local conduction delay in the computational model resulted in coved-type ST-elevation with negative T-waves in the precordial surface ECG leads. ‘Saddleback’-shaped ST-elevation was obtained with reduced substrate extent or thickness. Increased I_to_ simulations showed early repolarization in the RVOT with a descending but not coved-type ST-elevation. Reduced I_Na_ did not show a significant effect on ECG morphology.

**Conclusions:**

In this whole-heart BrS computational model of both major hypotheses, realistic coved-type ECG resulted only from delayed epicardial RVOT depolarization with local conduction delay but not early repolarizing ion channel modifications. These simulations provide further support for the depolarization hypothesis as electrophysiological mechanism underlying BrS.

## 1 Introduction

The Brugada syndrome (BrS) is an inheritable arrhythmia syndrome characterized by dynamic ST-elevations in right precordial leads and an increased risk of ventricular fibrillation (VF) and sudden cardiac death [6], [41]. The ST-elevation occurs in ECG leads located close to the right ventricular outflow tract (RVOT) but the pathology (channelopathy or mild cardiomyopathy) and the underlying mechanism (depolarization vs. repolarization disorder) causing ST-elevation and malignant arrhythmias are still controversial [16], [46], [43], [4]. The “repolarization hypothesis” states that in cases of reduced fast inward sodium current (I_Na_) abnormally early repolarization in the epicardial RVOT occurs due to a higher amount of unopposed transient outward potassium current (I_to_) and a loss of the epicardial action potential (AP) dome. The resulting dispersion of repolarization is theorized to create a substrate vulnerable to phase-2 reentry and would also result in a transmural gradient between endo- and epicardium during the plateau phase of the endocardium, manifesting as ST-elevation [16], [1]. The competing “depolarization hypothesis” on the other hand assumes an impaired conduction reserve in the epicardial RVOT with delayed conduction [26]. The latter hypothesis regards BrS not as a pure channelopathy but as a mild cardiomyopathy with micro-structural changes. It is supported by the following findings: (1) electrophysiological studies in BrS patients with epicardial RVOT recordings demonstrating low-voltage, highly fractionated electrograms (EGM) with very prolonged duration (up to >600 ms) [11] that are typical of slow conduction with (2) further prolongation and multi-site conduction block during extrastimulus pacing and/or sodium channel blocker administration [37], [31], [15] and (3) increased collagen content (myocardial fibrosis) in BrS patients RVOT epicardium [25], [31]. In addition, (4) catheter ablation of these abnormal EGMs in the epicardial RVOT has consistently led to ECG normalization and prevention of VF recurrence in highly symptomatic BrS patients [30], [29], [34], [32]. The presence of loss-of-function *SCN5A* mutations in around 20% of all BrS patients or presence of a rare non-coding *SCN5A* enhancer variant in up to 4% of Thai BrS patients that reduces I_Na_ up to 30% [2], [45] does not favor or eliminate either hypothesis.

Computational modeling has emerged as a powerful tool to study cardiac electrophysiology and arrhythmias [42] and has previously been used to shed light on the effects of either BrS hypothesis as no animal model recapitulates the entire BrS phenotype [39]. In addition, tissue-level computational studies have shown the possibility of malignant arrhythmias for both proposed mechanisms [10]. To compute realistic ECG leads, 3D whole-heart computational modeling of excitation conduction in the heart needs to be combined with field calculations in a torso model. Previous 3D simulations by Xia et al. [49] focused exclusively on the repolarization disorder hypothesis. Hoogendijk et al. [17], on the other hand, found that structural discontinuities needed to be combined with ion channel modulations to achieve significant ST-segment elevation – which was, however, horizontal, and not descending as would be expected for a typical coved-type BrS ECG. Here, we present a 3D whole-heart computational model of ST-elevation in BrS exploring both BrS disease hypotheses independently with the objective of investigating which of these would result in typical coved-type BrS ECGs.

## 2 Methods

### 2.1 3D whole-heart computational model

**Fig. 1:**
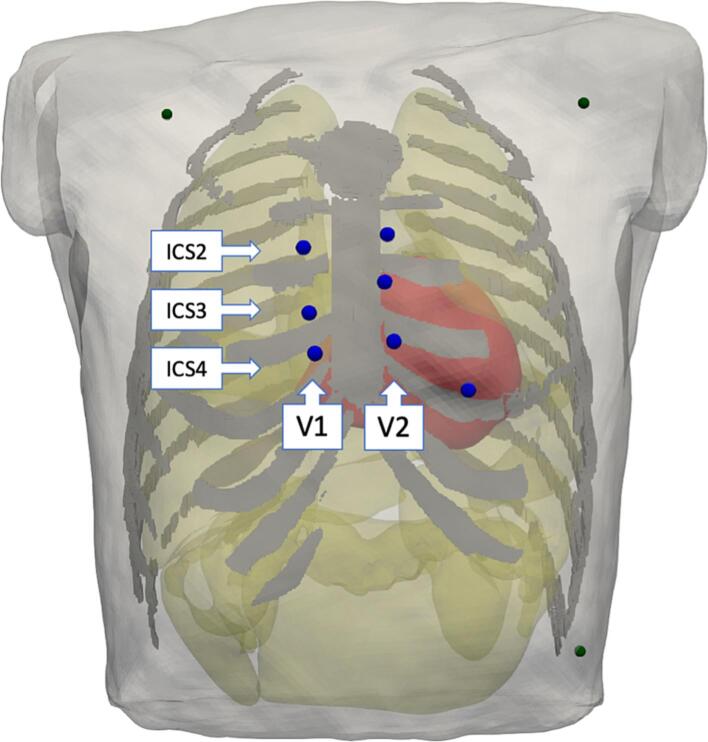
**3D torso model used for ECG calculation and simulated ECG tracings.** The ventricles are shown in red, organs in yellow, ribs in gray. The body surface ECG leads were placed parasternal right and left from the 2nd, 3rd, to 4th intercostal space (blue spheres). Smaller green spheres indicate locations of LA, RA, and LL electrodes used to compute the reference potential (Wilsons’s central terminal).

A 3D model of the heart and torso was adapted from a previous study in which MR images and a 64-lead ECG of a healthy 27-year-old male volunteer had been acquired [20], [27] (Fig. 1, Suppl. Video 1). The heart was newly segmented for this study as a tetrahedral mesh with a refined RVOT. The bi-ventricular mesh consisted of 1,262,315nodes (6,887,236 tetrahedra) with an average tetrahedron edge length of 0.65±0.13 mm (mean±SD). In the refined RVOT, edge length was 0.43±0.07 mm. The RVOT was refined as the area of interest for this study, while keeping the remaining mesh at a larger resolution to limit computational cost. The resolution-dependent difference in conduction velocity (CV) due to different resolutions was found to be negligible (see Supplementary Materials). The tetrahedral torso mesh was also refined allowing the newly segmented heart to fit seamlessly and to create a smoother body surface to reduce numerical errors in electrical field calculations. The average edge length of the torso mesh was 1.52 ± 1.23 mm with 4,609,725 nodes. Organ conductivities were set as stated in Table 1. Myocyte orientation (often referred to as fiber orientation) in the ventricles was set as described by Bayer et al. [3]. Myocyte orientation in the atria was set based on the approach described by Wachter et al. [44], even though no excitation conduction was computed in the atria. Nonetheless, myocyte orientation in the atria may influence the electric field during ventricular activity and therefore the ECG. The heart and torso meshes created for this study have been made publicly available [28].

**Table 1:** Conductivities used for lead field and ECG calculations.

Tissue	Conductivity (S/m)		Reference
Fatty tissue	0.035		Gabriel et al.[13], interpolated to 0 Hz using the Cole–Cole equation[8]
Skin	0.0002		
Blood *(only in heart cavities)*	0.7		
Lungs	0.03		
Intestines	0.01		
Kidney	0.05		
Liver	0.02		
Spleen	0.03		

Heart	*Longitudinal*	*Transverse*	
*Intracellular*	0.3	0.031525	Colli Franzone et al.[9]
*Extracellular*	0.15	0.05	Gabriel et al.[13], anisotropy from Keller et al.[19]

### 2.2 Computation of excitation, conduction, and electric potentials

To generate body surface ECG QRS complexes, simulations of excitation spread, and repolarization were conducted for the ventricles. Cellular electrophysiology was computed using the O’Hara–Virág–Varró–Rudy model of human ventricular myocytes [33]. The formulation of I_Na_ was modified to achieve nominal CV in tissue simulations as described in [12]. The endocardial variant of the model was used for the inner ¼ of the ventricular walls and the epicardial variant on the outer ¾, with the septum treated as if being an outer wall to the left ventricle. Ordinary differential equations defining the time course of this model were solved explicitly with a time step of 50 µs. The time course of transmembrane voltage distribution was computed using the monodomain model with the framework “acCELLerate” [38]. The framework has since been extended to run simulations on tetrahedral grids [14]. The finite element method was used to discretize the monodomain equation on the linear tetrahedral mesh. The monodomain equation was *integrated* using the Crank–Nicholson method with a time step of 50 µs, surface-to-volume ratio of 140.000 m^−1^, and specific membrane capacitance of 0.01 F/cm^2^. Monodomain myocardium conductivities were chosen as σ_t_ = 0.08 S/m in transverse and *σ*_l_ = 0.18 S/m in longitudinal direction, although it should be said that bulk monodomain conductivities may not correspond well to ‘physical’ conductivities because intra- and extracellular anisotropy ratios are, physiologically, not equal (which is an assumption underlying the monodomain model). The values resulted from adapting the duration of the simulated QRS complex in the healthy model to that of measurements from the volunteer.

The combination of the Crank–Nicholson method and using the full mass matrix (instead of ‘mass lumping’) allowed stable simulations with consistent CV at the spatial resolutions of our mesh (cf. [35], case *θ_lhs_*=0). We also took care to avoid ‘regular’ distributions of nodes in the mesh to achieve omnidirectionally CV [47]. Section S1 of the Supplementary Materials includes convergence simulations showing consistent simulated CV with the given conductivities up to the relevant spatial scale.

A lead-field matrix was used to calculate 64-lead surface ECGs from the resulting transmembrane voltages [36]. The lead field matrix was computed by solving the forward problem of electrophysiology once for each lead while a unit current was applied to that particular lead. The entries of the solution vector that correspond to intracardiac nodes then make up one row of the lead field matrix. Multiplying a vector of intracardiac transmembrane voltages with the lead field matrix results in a vector of potentials corresponding to the leads. Temporal resolution for ECG calculations was 1 ms. The simulated ECG lead placement on the torso model is shown in Fig. 1.

Sites of early activation in the ventricles were stimulated by a transmembrane current to mimic the Purkinje system. Location and timing of these stimuli were optimized using the method of Kahlmann et al. [18] with a 64-channel ECG of the volunteer as reference. Precordial leads and leads contributing to Wilson’s central terminal were weighted 3x during optimization. The optimization resulted in 225 stimulation sites in both ventricles over a time span of 149 ms.

Additionally, a gradient of maximal conductance of the slow rectifying potassium channel (*G*_Ks_) was applied such that *G*_Ks_ increases 3-fold from apex to base. This slightly shortens the AP towards the base (shortening APD_90_ by 10%), contributing to a (QRS-)concordant simulated T-wave [21], [23]. The above-mentioned transmural model variant difference was another major contributor to the T-wave morphology. The initial conditions of state variables for the 3D simulations were computed by first running individual cell models (clustered by similarity of parameters with each cluster comprising all cells where parameters differed less than 10%) until they reached steady states. These single-cell steady states were assigned to nodes in the 3D simulation. 4 beats where then computed in the 3D simulation and the 5th beat used as result.

Simulations were run on a rack-based high-performance computer featuring 2 AMD EPYC 7H12 64-core processors and 1024 GB RAM. Using 64 threads, computation of one electrophysiological cycle of 800 ms took approximately 45 min and computing a 64-lead lead field matrix took approximately 20 min. Optimization of the activation sequence was performed over 7 days using 128 threads.

### 2.3 Modelling of the epicardial RVOT substrate in Brugada syndrome

#### 2.3.1 Delayed depolarization model

**Fig. 2:**
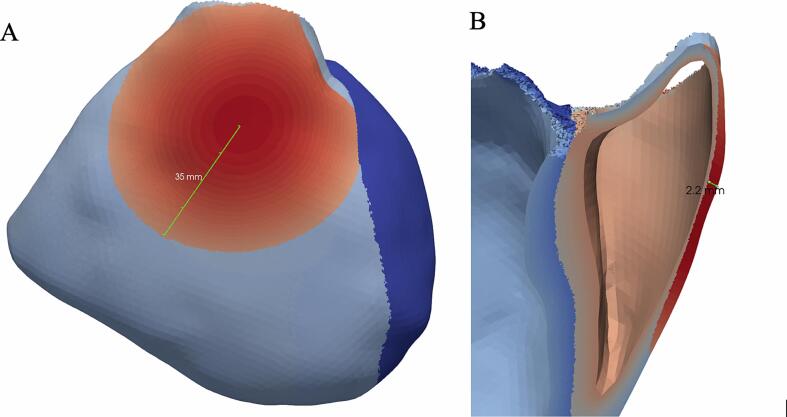
**Location and extent of modified substrate in the epicardial RVOT.** Conductivity was reduced in the substrate highlighted in red to simulate slow impulse propagation and delayed depolarization. The same region was used to simulate early repolarization by increasing local *G*_to_. Ablation was simulated by removing cellular activity in this epicardial region so that it behaves electrically passive. **(A)** Location on the ventricular wall. The right ventricle in light blue, left ventricle in dark blue. **(B)** Cross-section of the ventricular walls showing a maximal substrate thickness of 2.2 mm.

A pathological substrate was defined in a thin, approximately cylindrically shaped epicardial layer of the RVOT (Fig. 2). Conductivity of the substrate was reduced, informed by observations of slow conduction and prolonged EGMs in BrS patients. Substrate conductivity in longitudinal direction (with respect to local myocyte orientation) was reduced concentrically towards the midpoint of the substrate, from 0.09 S/m at the outer edges of the substrate to 0.009 S/m at the center, interpolated linearly between them. In isotropic models of uniform conductivity, one would expect CV to be reduced approximately 1.4- to 4.5-fold from such a 2- to 20-fold change [7]. The degree of conductivity reduction was chosen such that local activation of the substrate was delayed approximately as much as seen in BrS patients [22]. The concentrical pattern was inspired by observations by Pappone and Brugada et al. (2017), describing an “Onion-like” electrophysiological substrate with the longest potential duration in the centre of the RVOT. Substrate conductivity in transverse direction was attenuated 1000-fold, effectively dissociating the BrS region from more endocardially situated, normal conducting layers. A separate lead field matrix was computed for each model with reduced conductivity. However, only the intracellular conductivity was equivalently reduced for the lead field matrix computation – extracellular conductivity is here assumed to not be significantly affected by the remodelling that causes delayed depolarization.

In additional simulations, fast sodium channel conductance *G*_Na_ was reduced globally by 50% to simulate genetic predispositions, e.g., *SCN5A* loss-of-function mutations known to be found in around 20% of BrS patients, or the effect of sodium channel blockers like Ajmaline that are commonly administered to unmask a BrS ECG pattern. The 50% reduction of fast sodium current density is an assumption that may exceed experimental values [45].

#### 2.3.2 Early repolarization model

To simulate early repolarization, we increased maximal conductance of the transient outward potassium channel (*G*_to_) in the cellular model only in cells of the defined BrS substrate in the RVOT. This method has been used in a previous computational model of the repolarization hypothesis, albeit with a different ventricular cell model [49]. In additional simulations, *G*_Na_ was simultaneously reduced globally by 50% as described in the previous paragraph.

## 3 Results

### 3.1 Simulation of delayed depolarization through locally reduced tissue conductivity

**Fig. 3:**
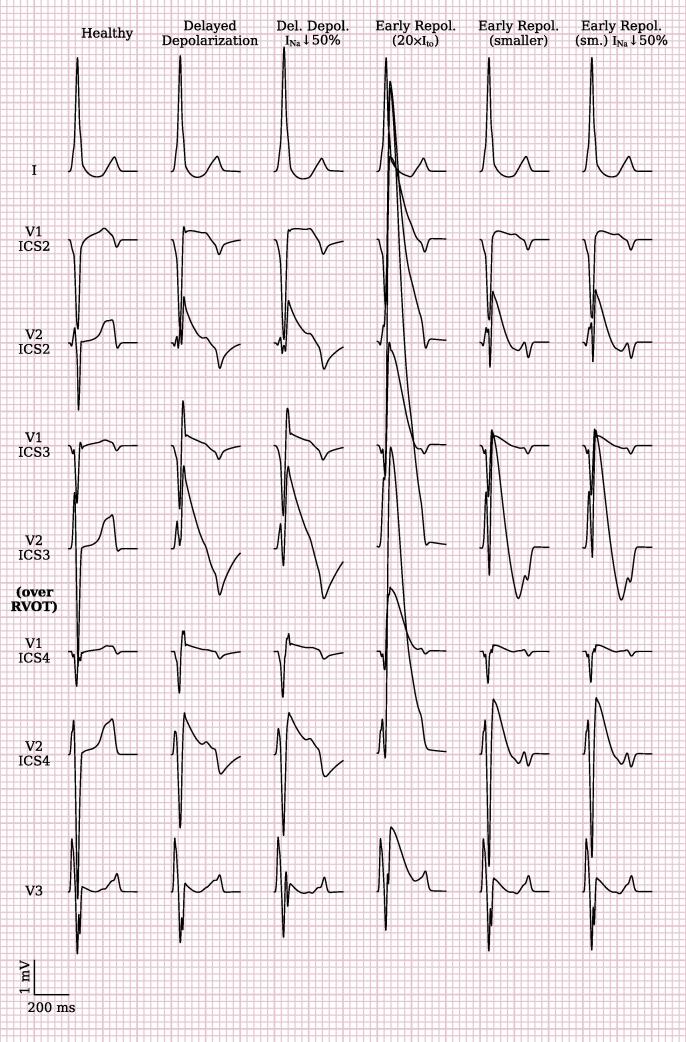
Simulated ECG tracings. “Healthy” variant has no substrate modifications. Conduction is locally slowed in “delayed depolarization” variants in the substrate labelled in Fig. 2. “Early repolarization” variants feature a 20-fold increase of G_to_. “Na↓” simulations include fast sodium channel loss-of-function by reducing its maximal conductance by 50 %. “Smaller” variants reduce the substrate extent to a diameter of 20 mm. Elevated J-points and ST-segment elevations can be seen in most simulations. With leads placed over the RVOT in the 3rd intercostal space (ICS), coved-type BrS ECG pattern is best visible (red circle).

Reducing conductivity in a thin epicardial RVOT layer (∅35 mm, thickness max. 2.2 mm) resulted in slow impulse propagation and thus delayed epicardial RVOT activation (Suppl. Video 2). ECG traces were evaluated in standard and high precordial lead positions with V1 and V2 simulated from the 2nd to the 4th parasternal intercostal spaces (Fig. 3, column “delayed depolarization”). Typical coved-type ECG shapes with descending ST-elevation (red circle) were observed only in the 3rd intercostal spaces (ICS) with the highest J-point elevation in the left parasternal ICS directly overlying the RVOT. Reduced I_Na_ slightly led to a slightly increased and more ‘convex’ ST-elevation (blue circle) (Fig. 3, column “Del. Depol. + I_Na_↓”). We also noted more prominent *secondary* repolarization abnormalities after reduction of I_Na_ like more negative T-wave amplitude and prolonged T-wave duration.

### 3.2 Influence of epicardial RVOT substrate extent and thickness on ECG morphology

**Fig. 4:**
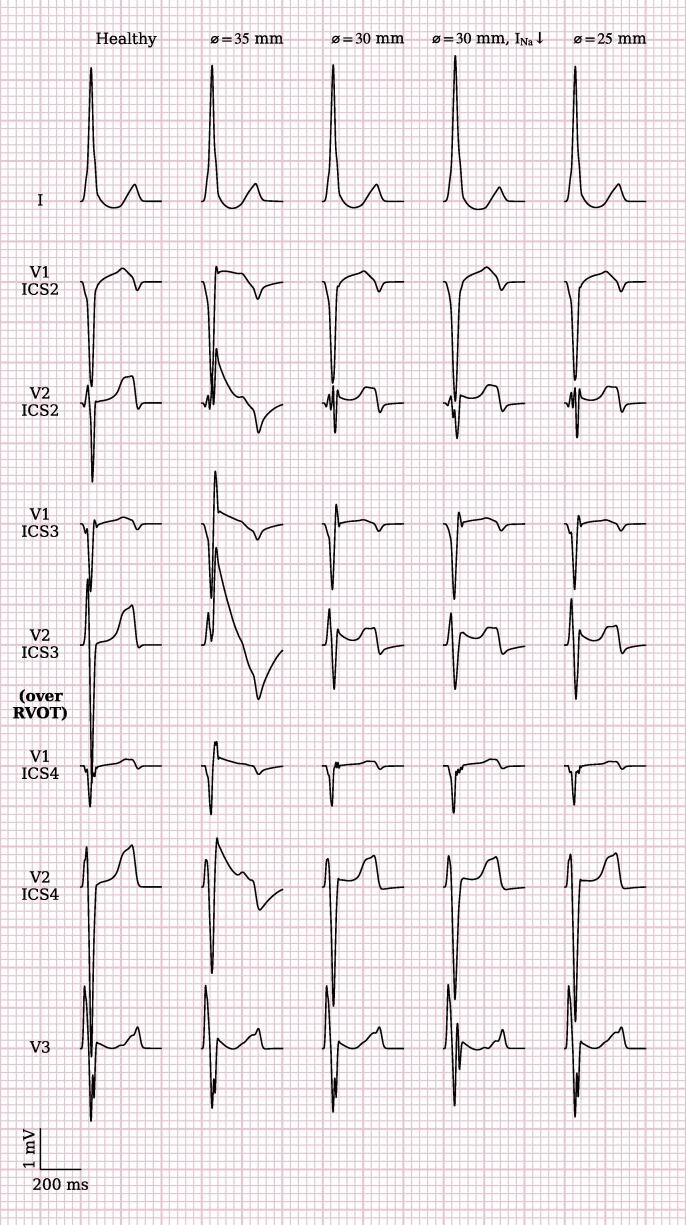
**Effect of substrate extent on ECG morphology.** Comparison of ECGs for different substrate diameters. Substrate center and thickness are unaltered compared to Fig. 2A and B, respectively, but diameter is reduced in subsequent columns. Reducing the substrate extent leads to conversion of coved-type to ‘saddleblack’ pattern ECG in the lead facing the RVOT (red and blue circles).

Multiple simulations with varying substrate size were performed. The diameters of the substrate on the epicardium were first varied without varying substrate thickness. In these simulations, reducing the diameter such that not the whole RVOT was covered resulted in conversion of coved-type to ‘saddleblack’ pattern ECG in the lead facing the RVOT (red and blue circles) (Fig. 4). Reduced I_Na_ (maximal conductance reduced to 50%) did not result in significant changes to the ECG.

**Fig. 5:**
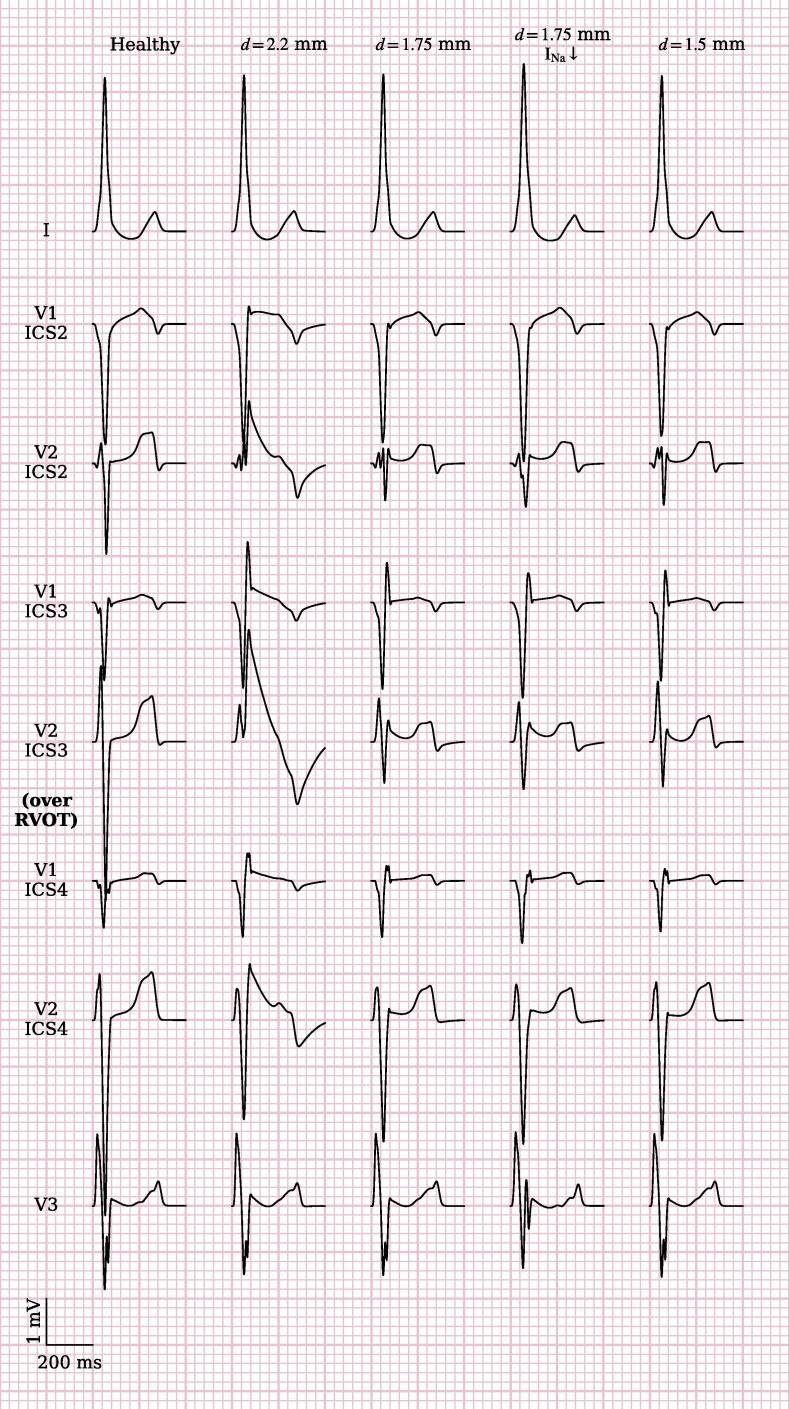
**Effect of substrate thickness on ECG morphology.** Comparison of ECG shapes for different substrate thicknesses. Substrate thickness is given in mm and refers to the thickest location. The center and diameter of the substrate are unaltered compared to Fig. 2A. Reducing the substrate thickness leads again to conversion of coved-type to saddleblack ECG in the lead facing the RVOT (red and blue circles) with lesser ST-elevation as the thickness becomes smaller.

Reducing substrate thickness resulted in again in conversion of coved-type to ‘saddleblack’ pattern ECG in the lead facing the RVOT (red and blue circles) (Fig. 5). Reduction of fast sodium channel conductivity did again not have a significant influence on the resulting ECG pattern.

### 3.3 Simulations of early repolarization through increased transient outward current

**Fig. 6:**
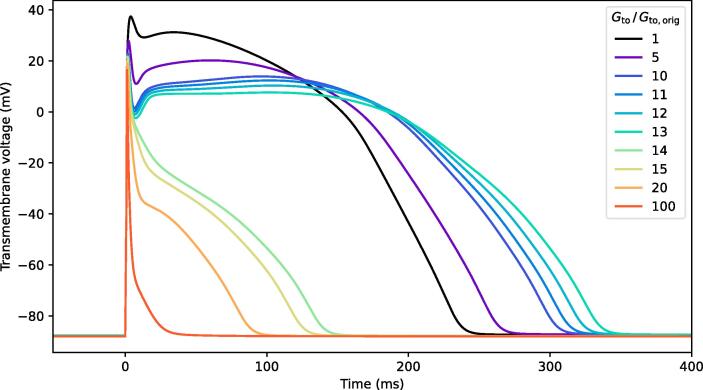
**Single cell action potential shapes for increased I_to_.** Simulated single cell action potentials (AP) of the O’Hara-Virag-Varro-Rudy human ventricular myocyte model. Initially, increasing I_to_ maximal conductance G_to_ results in prolonged AP. A 14-fold or higher increase results in a spikey morphology without plateau or dome, and early repolarization.

To achieve loss-of-dome in the single-cell AP model, *G*_to_ had to be increased at least 14-fold (Fig. 6), by which point the AP features no more plateau and its duration at 90% repolarization shortened to 132 ms. In the whole-heart simulations, a 20-fold increase was necessary to achieve early repolarization. When affecting the same region as in the simulations of delayed depolarization with delayed conduction, the resulting J-point amplitude was much higher (delayed depolarization: 2.4 mV in V2-ICS3early repolarization: 9 mV). Reducing the substrate thickness reduced the J-point amplitude but a comparable amplitude to delayed depolarization could not be achieved. The closes result was achieved at a substrate diameter of 20 mm and thickness of 0.88 mm, the J-point in V2-ICS3 was 3.5 mV (Fig. 3).

The resulting V2 leads still featured a more concave shape than with delayed depolarization, but V1 leads showed a linearly downward sloping ST-elevation. T-waves were negative in 3rd and 4th ICS, but wavy. Early repolarization simulations also show an elevated J-point of 0.6 mV in V3, unusual for BrS.

At substrate thicknesses below 0.88 mm, a 20-fold *G*_to_ increase was no longer sufficient to achieve early repolarization. As with delayed depolarization, a global reduction of *G*_Na_ to 50% only slightly changed the J-point and T-wave amplitudes in the early repolarization simulations (Fig. 3).

## 4 Discussion

A 3D whole-heart computational model – informed by delayed depolarization in a symptomatic BrS patient [22] – was created to test the main competing hypotheses of BrS. The presented simulations resulted in realistic coved-type ECGs, with the highest J-point elevation in the parasternal left intercostal space (corresponding to V2 in the 3rd ICS) directly overlying the RVOT. Importantly, no pathological ion channel changes have been made in this model of delayed depolarization and the repolarization abnormalities observed (more negative T-wave amplitude and longer T-wave duration in right precordial leads) were *secondary* to changes in depolarization. While we achieved delayed depolarization in the model by reducing conductivity in the RVOT, we do not suggest that this is the underlying mechanism. Reduced CV in the RVOT probably results from micro-structural changes, such as fibrosis plus a reduction of I_Na_ disrupting the propagation of excitation.

Even though conductivity was reduced linearly concentrically, the thinness of the layer of reduced conductivity combined with local myocyte orientation gave rise to a ‘sparkly’ pattern of activation (cf. Suppl. Video 2). Due to our relatively coarse mesh, this sparkly pattern may be considered an artefact rather than a result, although such patterns can be observed in EP mapping of BrS substrate.

A previous whole-heart computational modeling study, assuming the repolarization hypothesis as underlying mechanism, was able to reproduce Brugada-like ECG patterns in a simpler model of more coarse resolution and isotropic torso conductivities [49]. We attempted to reproduce their results and modified our model accordingly by increasing *G*_to_. The resulting ECGs display some BrS characteristics. However, we were unable to achieve a coved-type ECG in V2 leads with this method. Negative T-waves remained wavy and including I_Na_ loss-of-function was without significant effect on the ECG.

Anatomically, our model is limited by resolution. No micro-structural discontinuities were thus included in our simulations of the depolarization hypothesis and only reduction of conductivity in the RVOT was used to slow the local activation and conduction velocity. Introduction of such discontinuities has been done previously by Hoogendijk et al. [17] but resulted in ST-elevation only when combined with modulations of I_to_ and/or I_CaL_ and even then, the elevated ST-segment was horizontal and not descending as would be expected in a coved-type BrS ECG pattern. It is not clear what causes this discrepancy – pilot simulations of structural discontinuities in our model showed non-diagnostic descending ST-elevation that increased in amplitude with added discontinuities (preliminary data not shown). However, complete conduction block occurred before ST-elevation became diagnostic. With complete conduction block, the resulting ST-elevation was horizontal and not descending. We hypothesize that a higher resolution model with more discrete discontinuities resulting in only regional conduction block along with slow impulse propagation would produce similar ECGs as seen in our slowed conduction model.

In summary, in this whole-heart BrS computational model of both major hypotheses, realistic coved-type ECG resulted *only* from delayed conduction and slow impulse propagation in the epicardial RVOT but not early repolarizing ion channel modifications. Together with the known clinical observations in BrS patients, this simulation study provides further support for the depolarization disorder hypothesis as electrophysiological mechanism underlying ST-elevation in BrS. BrS-pattern ECGs were not achieved in early repolarization simulations. Our simulations also confirm that high precordial lead placement is necessary to detect a coved-type BrS ECG with the strongest BrS patterns found in the 3rd ICS. In the future, more complex computational models should incorporate micro-structural discontinuities, the genetic alterations found in individual BrS patients and modulations of the vagal tone to assess the arrhythmogenicity of the BrS substrate and refine risk stratification.

## 5 Limitations

As our pattern of reduced conductivity is concentric and evenly distributed, no zig-zag conduction and sites with local conduction block were observed as impluse propagation was slowed and depolarization occurred delayed but mostly evenly. Therefore, our epicardial EGMs were prolonged but not fractionated which is a key electrophysiological feature of BrS pathogenesis and arrhythmogenesis. Importantly, sites of fractionation with decremental properties are prone to unidirectional conduction block facilitating re-entry [5], [40]. Notably, our simulations barely show any effect of reducing fast sodium channel conductance, even when halving it. Higher resolution micro-structural simulations (including fibrotic discontinuities) will likely be more affected by such changes due to the less even distribution of electric load.

The QRS morphology in V1–V3 of the simulated ECGs are not entirely realistic and can be improved by Purkinje tree adaption based on 64-channel ECG recordings of the volunteer [18], [27]. ECG amplitudes may be larger than expected as not all sources of attenuation are included in the simulation (e.g., subdermal skeletal muscle layers [24] and selected tissue conductivities may be not sufficiently accurate.

## 6 Conclusion

The presented 3D whole-heart computational model based on the depolarization disorder hypothesis recapitulates major features of ST-elevation in BrS: (1) ST-elevation found in proximity to the RVOT substrate with (2) augmentation and (3) conversion of a type-2 to type-1 ECG by a reduction of I_Na_. Thus, our results support the depolarization disorder hypothesis as the main mechanism underlying BrS.

## 7 Grant support

EMW is a member of collaborative research center SFB1425, funded by the Deutsche Forschungsgemeinschaft (DFG, German Research Foundation, project no. 422681845). The work of EMW and GS was partially funded by DFG under project no. 183027722.

## Funding

EMW is a member of collaborative research center SFB1425, funded by the Deutsche Forschungsgemeinschaft (DFG, German Research Foundation, project no. 422681845). The work of EMW and GS was partially funded by DFG under project no. 183027722.

## Data availability statement

3D meshes from this study are available as an open-access dataset [28]. Electrophysiological simulation results are also available [48]. Any other data underlying this article will be shared upon reasonable request to the corresponding author without undue delay.

## CRediT authorship contribution statement

**Eike M. Wülfers:** Writing – review & editing, Writing – original draft, Visualization, Software, Methodology, Investigation, Formal analysis, Data curation, Conceptualization. **Robin Moss:** Writing – review & editing, Visualization, Data curation. **Heiko Lehrmann:** Writing – review & editing, Validation. **Thomas Arentz:** Supervision. **Dirk Westermann:** Supervision. **Gunnar Seemann:** Writing – review & editing, Supervision, Software, Resources, Project administration, Funding acquisition, Conceptualization. **Katja E. Odening:** Writing – review & editing, Supervision, Conceptualization. **Johannes Steinfurt:** Writing – review & editing, Writing – original draft, Validation, Supervision, Investigation, Conceptualization.

## Declaration of competing interest

The authors declare that they have no known competing financial interests or personal relationships that could have appeared to influence the work reported in this paper.
