# Supplementary material for: Whole-heart computational modelling provides further mechanistic insights into ST-elevation in Brugada syndrome
Source: Int J Cardiol Heart Vasc. 2024 Mar 4;51:101373. doi: 10.1016/j.ijcha.2024.101373 (PMC10924145; doi:10.1016/j.ijcha.2024.101373)
Supplement: Supplementary data 3 [file mmc3.pdf]

Supplementary Material

1 Convergence Simulations

Using the monodomain parameters stated in the paper and the same cellular electrophysiology model, excitation was simulated in a cuboid geometry as used in the electrophysiology simulation software benchmark by Niederer et al. (2011). The cuboid mesh was generated using the software Gmsh (Geuzaine & Remacle, 2009), which we previously found to create meshes with a sufficiently quality, particularly distribution of nodes avoiding preferential directions of conduction (Wülfers et al., 2016).

Activation times were measured in 100 equidistant locations along the diagonal from the corner of stimulation to the opposite corner. Activation times were linearly interpolated from element vertices when such locations did not coincide with a mesh node. Supplementary Figure 1 displays the activation times measured along the diagonal for the tested resolutions. CV was determined along this diagonal (i.e., not parallel to fibre orientation), only considering values at least 3 mm from the corners. The results are shown in Supplementary Table 1.

Notably, the CV does not monotonically decrease with finer resolution: the CV at 0.1 mm is slower than at 0.05 mm. This observation is in line with previous independent studies using comparable numerical methods, specifically the fully populated mass matrix, when solving the partial differential equation (Pezzuto et al., 2016; Niederer et al., 2016 (Software 'A')).

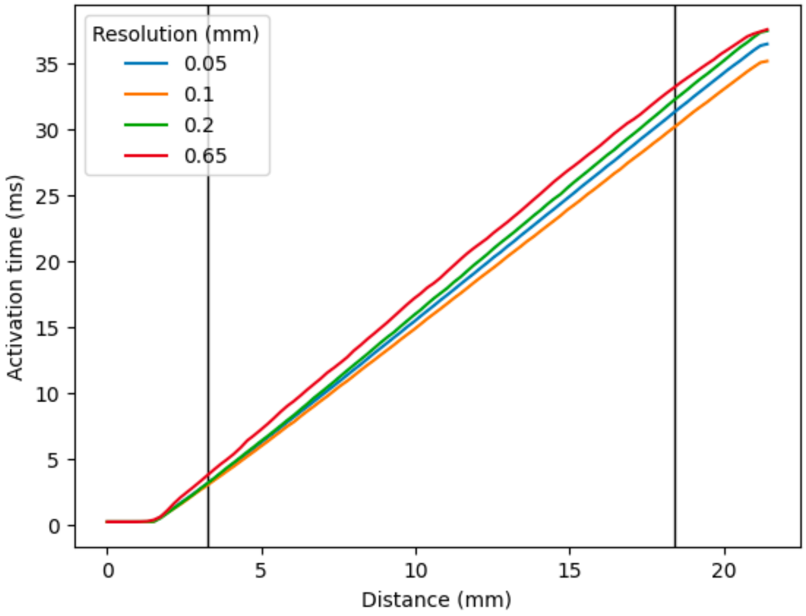

Supplementary Figure 1: Activation times along diagonal axis of the simulated cuboids for different tetrahedral resolutions. Black delimiters show limits between which CV was computed as average derivative.

Supplementary Table 1: Conduction velocities (CV) along the diagonal and activation times of the far corner node for different resolutions from Supplementary Figure 1.

Resolution (mm, ±SD)	CV (mm/s)	Activation time of far corner (ms)
0.05 ± 0.01	538.55	36.45
0.1 ± 0.02	558.85	35.15
0.2 ± 0.05	521.96	37.45
0.65 ± 0.18	517.51	37.55

2 Resolution effects

The elements of the RVOT of the whole-heart model were refined for this work, resulting in an average tetrahedral edge length of 0.43 mm there and 0.7 mm elsewhere (0.65 mm average overall). Supplementary Figure 2 shows the tetrahedra at the boundary between the two regions.

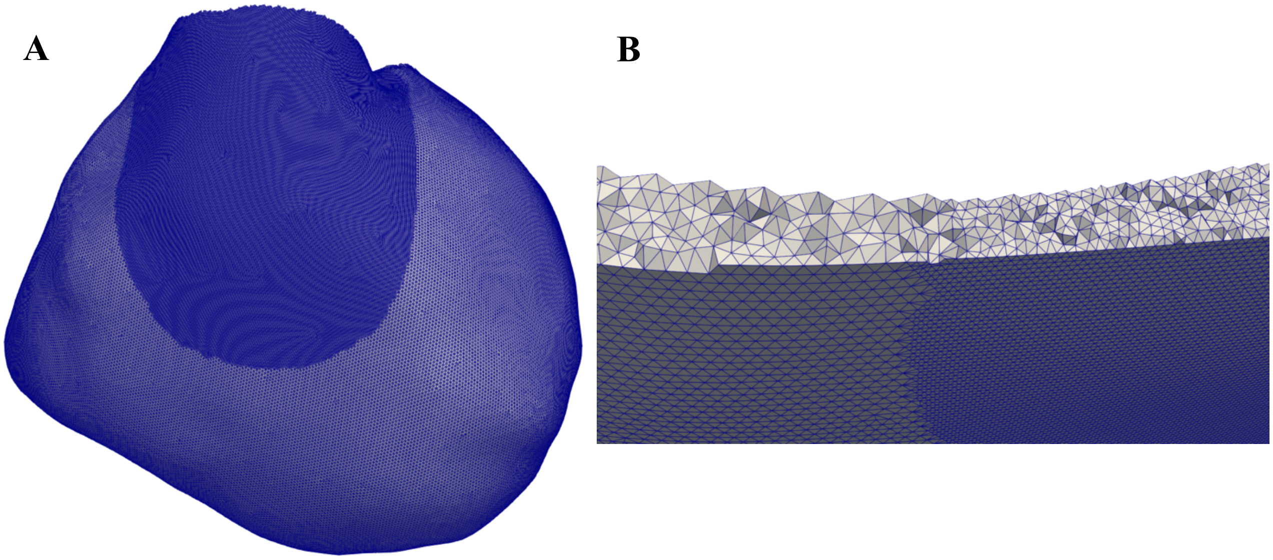

Supplementary Figure 2: Simulation mesh with tetrahedra edges in blue. A) View of the RVOT. B) Cut through the right ventricular wall such that tetrahedra from the coarse and the fine segments of the mesh are visible.

To assess effects possibly caused by the sudden change in resolution between RVOT and the rest of the mesh, we measured CV in three benchmark simulations. Two simulations were performed in a small tetrahedral grid with the conduction direction perpendicular to the resolution boundary (i.e., the wavefront parallel to the resolution boundary, Supplementary Figure 3A). One simulation was performed changing resolution from coarse (0.7 mm) to fine (0.43 mm), the second simulation in reverse direction. Activation times were measured along the central axis of the mesh, parallel to the direction of conduction. The third simulation was performed with the direction of excitation parallel to the resolution boundary. Here, we measured activation times in both, the coarse and fine region of the mesh.

All simulations showed an effect of the resolution change on the CV of 10% slowdown from coarse to fine, which was negligible compared to the imposed CV change in our simulations and comparable to CV errors that would occur e.g. due to imperfect fibre orientations. Importantly, no sudden effects or instabilities were observed in these simulations.

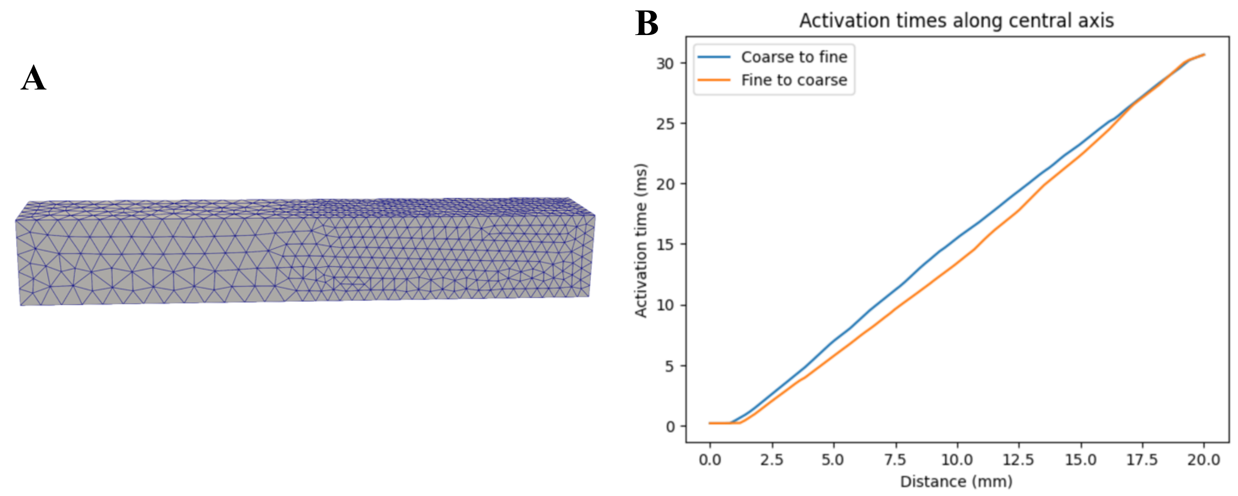

Supplementary Figure 3: A) Benchmark mesh with tetrahedra edges in blue. B) Activation times along the central longitudinal axis for a simulation from coarse to fine (blue) and from fine to coarse (orange).

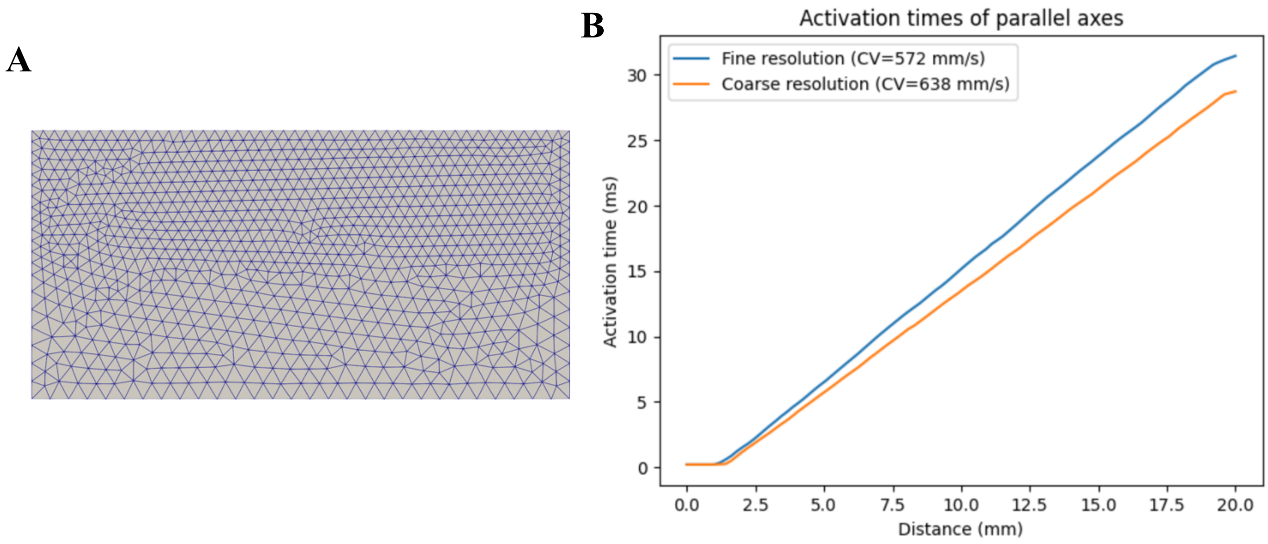

Supplementary Figure 4: A) Benchmark mesh with tetrahedra edges in blue. B) Activation times along longitudinal central axes through the coarse (blue) and fine (orange) segment of the mesh.

3 Effects of I_{Na} blocking

Unfortunately, the presented simulations were not sensitive to partial block of I_{Na}. Our investigations showed that the fast sodium current in the Dutta et al.-modified O'Hara–Virag–Varro–Rudy-model

was very strong: in single cell mode, the model produced AP even at 97.5% block of I_{Na} (with the maximum upstroke velocity (dV/dt) reduced from 552 V/s to 30 V/s, Supplementary Figure 5). A stimulation current of 80 pA/pF was only applied until the model reached –40 mV transmembrane voltage and injected every 800 ms for 100 s. The final complete beat was analysed.

In tissue simulations, we observed a similar sturdiness, a 90 % block of I_{Na} simply reducing CV from 670 mm/s to 352 mm/s (52%, Supplementary Figure 6) – negligible compared to the CV reductions we imposed through conductivity reductions. Therefore, in the absence of microstructural components imposing significant additional load to the simulated cells, any I_{Na} blocking in this model would only slightly affect conduction velocity in the BrS substrate. (Simulations were carried out in a geometry like in Supplementary Figure 3A, but with a uniform resolution of 0.4 mm).

The strong I_{Na} can also not easily be overcome by I_{to} increases, as shown in Supplementary Figure 7 (varying degrees of Ito increase at 50% I_{Na}) which is virtually identical to Figure 6 of the main manuscript (at 100% I_{Na})

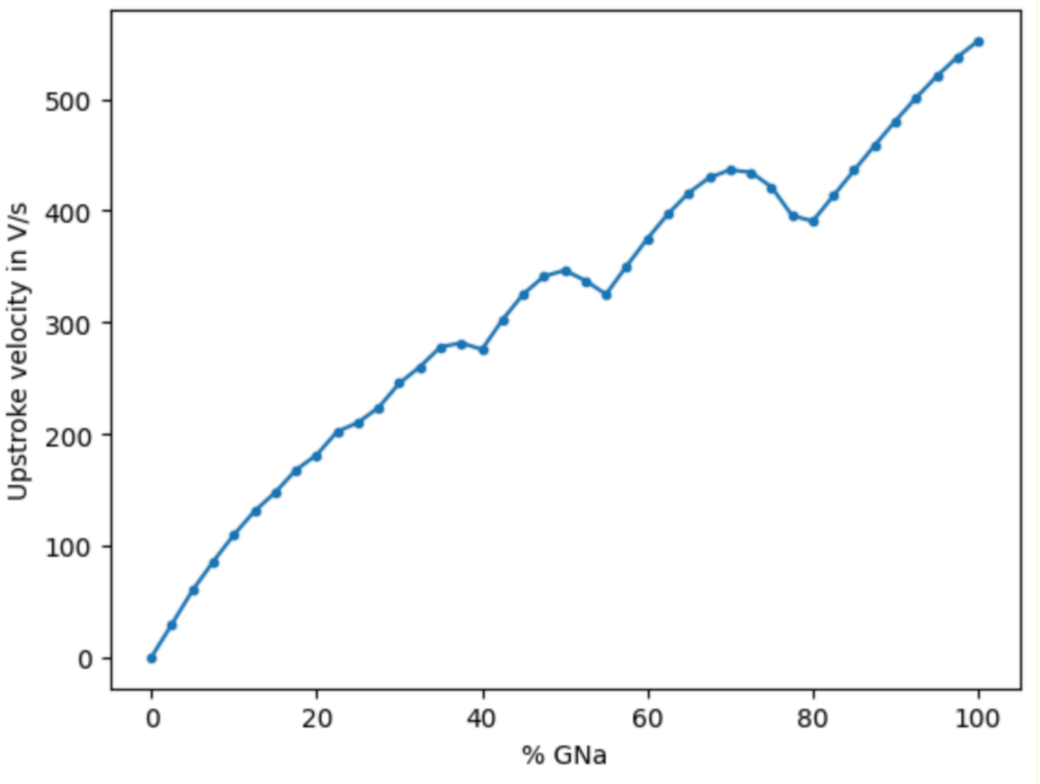

Supplementary Figure 5: Upstroke velocity versus fast sodium channel conductance G_{Na} in single cell mode.

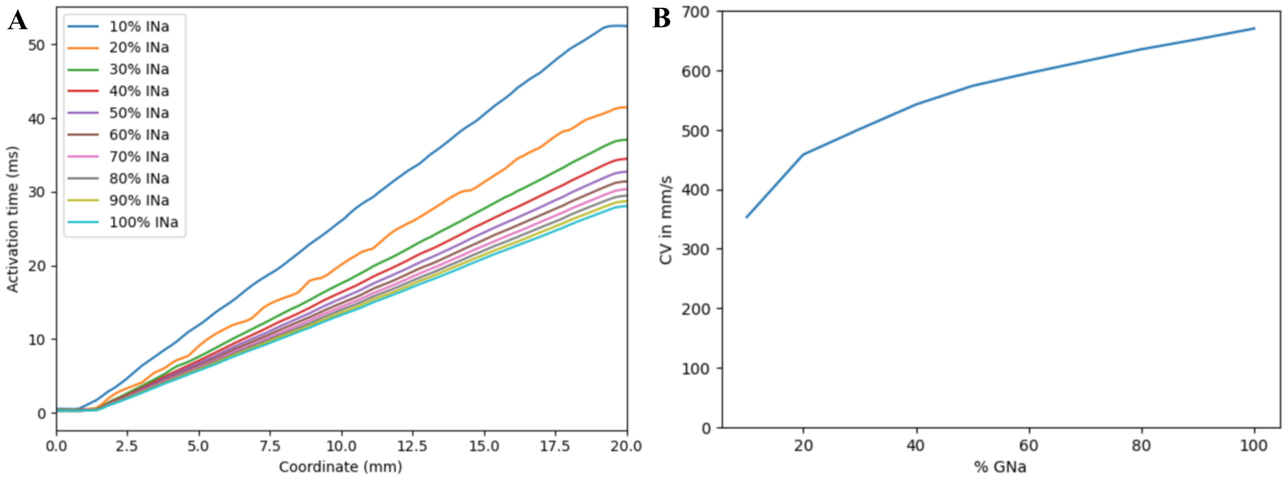

Supplementary Figure 6: A) Activation times along central diagonal of a benchmark mesh vor varying degrees of I_{Na} block. B) Resulting conduction velocities over remaining G_{Na}.

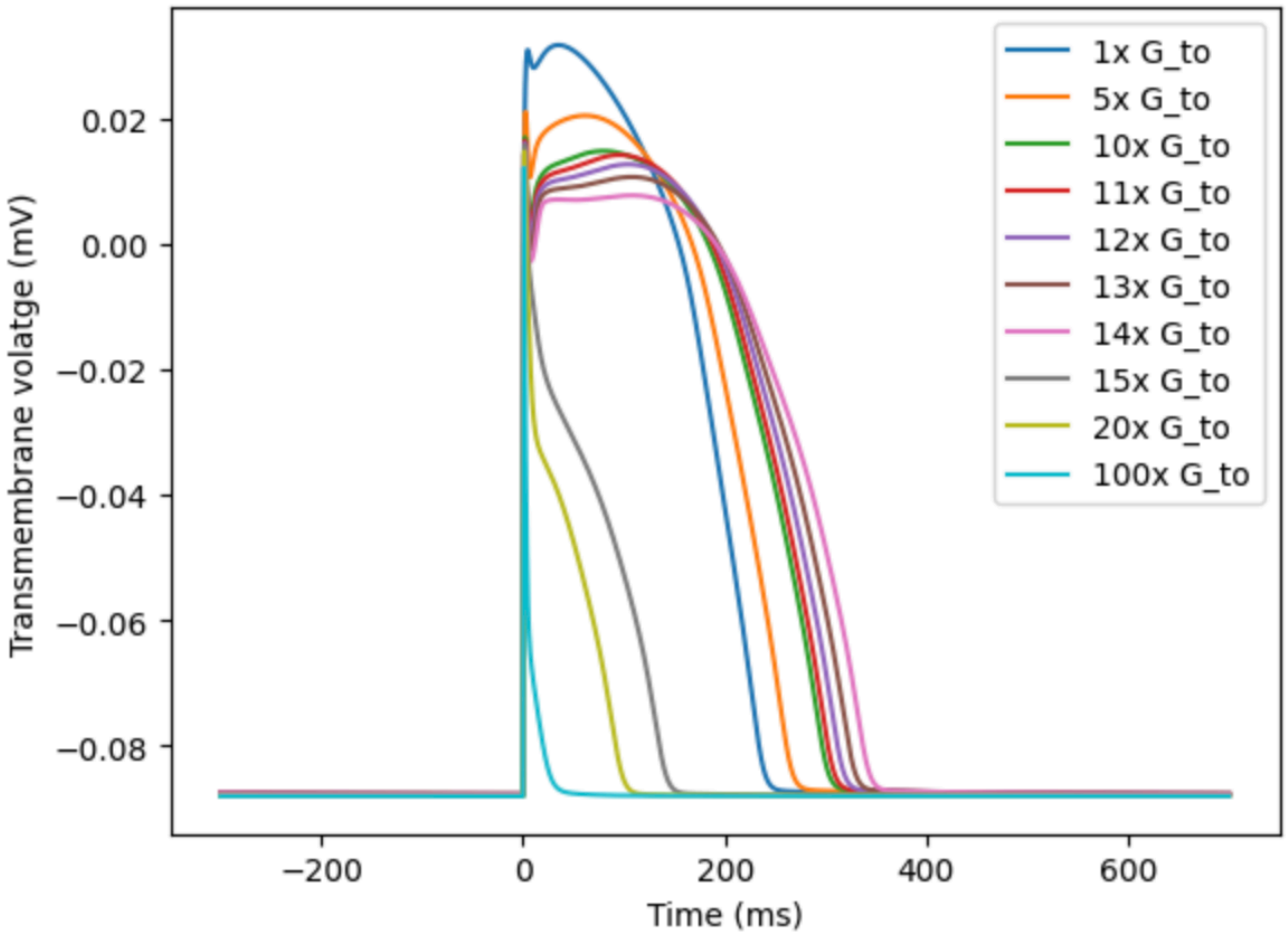

Supplementary Figure 7: Action potentials with varying degrees of I_{to} increase at 50% I_{Na} block. Compare with figure 6 of the main manuscript for plots at 100% I_{Na}.

4 References

Geuzaine, C. & Remacle, J.-F. 2009. Gmsh: a three-dimensional finite element mesh generator with built-in pre- and post-processing facilities. *International Journal for Numerical Methods in Engineering* **79**(11), 1309–1331.

Niederer, S. A., et al. 2011. Verification of cardiac tissue electrophysiology simulators using an *N*-version benchmark. *Phil. Trans. R. Soc. A*. **369** 4331–4351. http://doi.org/10.1098/rsta.2011.0139

Pezzuto, S., Hake, J. & Sundnes, J. 2016. Space-discretization error analysis and stabilization schemes for conduction velocity in cardiac electrophysiology. *International Journal for Numerical Methods in Biomedical Engineering,* 32(10), e02762.

Wülfers, E. M., Dössel, O. & Seemann, G. 2016. Regularity of node distribution impacts conduction velocities in finite element simulations of the heart. *2016 Computing in Cardiology Conference (CinC)*, Vancouver, BC, Canada, 2016, 177–180.
